# 30 Oligoarticular juvenile idiopathic arthritis-associated uveitis

**DOI:** 10.1093/rheumatology/keac496.026

**Published:** 2022-10-07

**Authors:** Rahma Guedri

**Affiliations:** Children Hospital Tunisia

## Abstract

**Background:**

Oligoarticular juvenile idiopathic arthritis (JIA) is a rare inflammatory disease that occur in children under the age of 16. JIA associated uveitis is the most frequent extra-articular manifestation. The uveitis can be sight-threatening and may be burdened with disabling morbidity. The uveitis seen in JIA is chronic anterior uveitis which is always asymptomatic in the initial stages.

**Aim:**

The purpose of our survey is to report the incidence of uveitis in oligoarticular JIA and to determine the potential risk factors for the occurrence of uveitis in children with oligoarticular JIA.

**Results:**

We retrospectively evaluated 70 consecutive pediatric patients with oligoarticular JIA in the emergency and outpatient paediatric departement at the children's hospital in Tunisia between January 2005 and December 2021. A total of 12 cases (17.1%) with oligoarticular JIA associated uveitis where identified. The incidence of uveitis in these children was 0.7 cases per year.

The uveitis had occurred before the joint manifestations in only one patient. The average time between the onset of symptoms and the occurrence of uveitis was 1.7 years with a maximum time interval of 5 years. Management of JIA-associated uveitis involved use of topical agents in half of our patients and systemic agents in the other half.

JIA-associated uveitis has led to ocular complications such as cataracts (*n* = 5), glaucoma (*n* = 6), anterior/posterior synechiae (*n* = 8), and ultimately a visual impairment and blindness (*n* = 1). Eleven of the twelve patients with uveitis retained acceptable visual acuity.

The presence of antinuclear antibody (ANA) has been identified as a risk factor for the occurrence of uveitis (*p* = 0.03).

**Conclusion:**

Collateral damage of oligoarticular JIA include growth failure, muscle atrophy and intraocular damage. The main challenge during the management of oligoarticular JIA is the early detection of uveitis. Close cooperation between the rheumatologist and the ophthalmologist is essential to optimize outcome.

